# The Holm and Cordoba Urinary Tract Infection Score: Translation, Linguistic and Content Validation of the German Version of a Patient‐Reported Outcome Measure to Assess Symptoms, Bothersomeness and Impact of Uncomplicated Urinary Tract Infections in Women

**DOI:** 10.1002/nau.70066

**Published:** 2025-05-08

**Authors:** Katharina Piontek, Sophie Nestler, Ebru Özkan, Anne Holm, John Brandt Brodersen, Christian Apfelbacher

**Affiliations:** ^1^ Institute of Social Medicine and Health System Research Otto‐von‐Guericke‐University Magdeburg Magdeburg Germany; ^2^ The Centre of General Practice University of Copenhagen Copenhagen Denmark; ^3^ The Research Unit for General Practice, Region Zealand Copenhagen Denmark; ^4^ Department of Community Medicine, Faculty of Health Sciences, Research Unit for General Practice UiT The Arctic University of Norway Tromsø Norway

**Keywords:** content validity, COSMIN, patient‐reported outcome measure, qualitative interviews, urinary tract infections

## Abstract

**Background:**

The Danish Holm and Cordoba Urinary Tract Infection Score (HCUTI) assessing symptom severity, bothersomeness and impact of uncomplicated urinary tract infections (uUTIs) on daily activities in women is a promising patient‐reported outcome measure (PROM) for use in future research. For potential application in Germany, the present study aimed (i) to perform translation and linguistic validation of the HCUTI, and (ii) to assess content validity of the German version of the HCUTI.

**Methods:**

Translation and linguistic validation was performed using the dual‐panel method. A bilingual panel of nonprofessional Danish‐German speaking persons translated the HCUTI, and the translated version was evaluated regarding comprehensibility by a lay panel of native German‐speaking women with past uUTI. Content validity of the German version of the HCUTI was assessed according to the criteria of the COnsensus‐based Standards for the selection of health Measurement INstruments (COSMIN) methodology. In individual cognitive interviews, women with a history of uUTI and experts from different medical fields rated the instructions, items, response options and recall period of the HCUTI in terms of relevance, comprehensiveness and comprehensibility.

**Results:**

Translation and linguistic validation resulted in a German version of the HCUTI which lay persons considered easy to understand. In content validity assessments, participants rated the questionnaire as generally relevant and comprehensive. One item was removed due to lack of relevance. The response options were linguistically modified, and a dichotomous scale was introduced for 10 items on symptoms. To enhance comprehensibility, eight items on symptoms and bothersomeness, and one item on daily activities were slightly reformulated. After modification, the German version of the HCUTI includes 18 items on symptoms, 18 items on bothersomeness, and six items on impact on daily activities. The study team reviewed and linguistically standardized the final version of the questionnaire to ensure consistency in wording and sentence structure across all items.

**Conclusions:**

The German version of the HCUTI is a suitable tool to assess symptoms, bothersomeness and impact of uUTIs in women. Data on psychometric properties of the instrument will be collected in a subsequent survey among women with uUTIs.

## 1 Introduction

Uncomplicated urinary tract infections (uUTIs) in women are among the most common bacterial infections in the outpatient setting [1]. Although the condition is often self‐limiting, the majority of women with symptoms suggestive of uUTI are treated with antibiotics to achieve rapid symptom resolution [2]. However, the potential inappropriate and excessive use of antibiotics has led to resistant strains of uropathogens, which has significant implications for public health [3]. Given the health threats related to conservative antibiotic treatment, identifying adequate alternatives to antibiotics has high priority [4]. Further, women use a wide range of strategies in their self‐management of UTI symptoms such as herbal remedies [5]. Evaluating the effectiveness of these treatment strategies from the patient's perspective is critical, and patient‐reported outcome measures (PROMs) are valuable tools for this purpose. PROMs are standardized questionnaires for the assessment of health outcomes directly from the patient, for example, symptoms, functional status, treatment side effects and health‐related quality of life [6]. For use in women with uUTIs, several disease‐specific instruments have been developed and validated. The selection of an appropriate PROM is based on considerations regarding the construct to be measured, but also takes the quality of the measurement properties of available instruments into account. To guide this process, the COnsensus‐based Standards for the Selection of Health Measurement INstruments (COSMIN) initiative has developed guidelines, detailed criteria and practical tools for the identification of high‐quality PROMs [7]. In a previous systematic review of the quality of measurement properties of PROMs for women with uUTI following the guidelines of the COSMIN group, our research group identified six instruments measuring a variety of outcomes [8]. Among those, the Danish Holm and Cordoba Urinary Tract Infection Score (HCUTI) was found to capture the broadest spectrum of outcomes including symptom severity, bothersomeness, and impact on daily activities [9]. Our evaluation of measurement properties revealed that the HCUTI has the potential to be used in future clinical studies, but requires further validation (COSMIN category B) [8]. As a major strength of the HCUTI, patients were involved in the development of the instrument, meeting the requirements for PROMs of regulatory authorities such as the European Medicines Agency [10] and the U.S. Food and Drug Administration [11]. Also sufficient content validity in terms of relevance, comprehensibility, and comprehensiveness from the patient's perspective has been found.

For potential application in Germany, the present study pursued the following aims:

i. to perform translation and linguistic validation of the HCUTI and
ii. to evaluate content validity of the German version of the HCUTI

## 2 Materials and Methods

### 2.1 Translation and Linguistic Validation

#### 2.1.1 Study Design

Translation and linguistic validation were performed using the dual‐panel method [12]. As an alternative to the forward‐and‐back‐translation method, which is widely considered the “gold standard” for translating PROMs [13], the main focus of the dual‐panel method is on the consensus translation process involving non‐patient and patient lay persons [12]. Evidence suggests that this method has advantages over the forward‐and‐back‐translation method in terms of preference by lay people, but shows no obvious psychometric differences [14].

First, a bilingual panel of nonprofessional individuals fluently speaking Danish and German translated the questionnaire in a web‐based meeting. The translation process was guided by the study team, and all discussion points and decisions were documented in meeting minutes. To ensure that the meaning of all items was maintained, the developer of the HCUTI (AH) participated in the meeting and provided explanations if required. Second, the translated version of the questionnaire was reviewed for comprehensibility in another web‐based meeting by a lay panel of German native‐speaking women with a history of uUTI. This session was audio‐recorded for later analysis, and changes in wording were documented in meeting minutes. The research team adapted the questionnaire according to the findings from linguistic validation, and the resulting German version of the HCUTI was used for the assessment of content validity.

#### 2.1.2 Recruitment of Participants

Participants of the translation panel were recruited using a flyer which was distributed in universities, language academies and libraries in the German–Danish border region. Individuals at least 18 years old with sufficient Danish and German language skills (Danish/German native speakers, bilingual persons) were considered eligible. Participants of the translation review panel at least 18 years old were recruited via personal contacts of the study team.

### 2.2 Content Validation

#### 2.2.1 Study Design

Content validity was assessed following the criteria of the COSMIN group [15]. In single cognitive interviews, women with a history of uUTI and experts from relevant disciplines evaluated relevance, comprehensiveness, and comprehensibility of the items, instructions, response options and recall period according to a standardized semi‐structured interview guide (Supporting Information S1: Appendix A). Using a combination of the current think aloud methodology and targeted questions (Q‐by‐Q testing) [16], participants were instructed to read each item aloud, to articulate their thoughts aloud, and to rate comprehensibility and relevance of each item as follows: (1) “item is clear/relevant,” (2) “item needs minor revisions to be clear/relevant,” (3) “item needs major revisions to be clear/relevant,” or (4) “item is not clear/relevant.” Subsequently, the appropriateness of the response options and the recall interval, and comprehensiveness of the questionnaire were evaluated using open questions. All interviews were performed by a researcher (SN) trained in conducting qualitative interviews. Before the interviews, participants were briefed regarding study background, interview procedure and data protection regulations. The interviews were audio‐recorded and transcribed verbatim using MaxQDA (MAXQDA 2020.4.2, VERBI Software, 2024). Transcripts were reviewed and anonymized to ensure confidentiality. The interviews lasted about 1 h.

#### 2.2.2 Recruitment of Participants

The assessment of content validity according to COSMIN criteria involves a minimum of seven patients from the target group and seven experts from relevant disciplines. Study participants were recruited via the network of the study team. Women at least 18 years old with sufficient German language skills, who had experienced an uUTI within the last 3 years, were considered eligible for study participation. Experts from relevant disciplines including family practice, urology, gynaecology, nursing, health sciences, psychosomatic medicine, and research methodology were also recruited via the network of the study team.

#### 2.2.3 Materials

The Danish version of the HCUTI [9] is a 43‐item questionnaire to be completed as daily diary each night before going to bed. All items refer to the last 24 h, and are rated on a 4‐point Likert scale as none (0), a little (1), some (2) or a lot (3). The instrument comprises the following three domains:

a. Symptom severity (18 items)
b. Bothersomeness (18 items)
c. Impact on daily activities (7 items)

#### 2.2.4 Data Analysis

Transcripts were coded independently by two researchers (SN, EÖ) using Microsoft Excel sheets. Coding was performed for open questions concerning response options, recall period, and comprehensiveness. Data on relevance and comprehensibility were re‐coded if participants had not responded according to the numerical system during the interviews, or if the assigned code did not match the participant's comment. Potential modifications were discussed within the study team and by consulting the developer of the HCUTI (AH) if at least three participants rated an item as requiring at least minor revisions to be clear/relevant, thereby considering concrete statements of the participants. Overall, the comments of participating women were considered more important with respect to the need of potential modifications of the instrument than the comments of experts. Throughout this process, careful attention was paid to ensure that all modifications aligned with the initial version of the questionnaire without altering the intended meaning of the items. Where modifications were necessary, the main aim was to improve comprehensibility, relevance and comprehensiveness of the questionnaire. This iterative process continued until the questionnaire garnered acceptance from the majority of participants.

### 2.3 Ethics

The study was approved by the Ethics Committee of the Otto‐von‐Guericke University, Medical Faculty and University Hospital Magdeburg (No. 19/23). All study participants gave written informed consent. A financial incentive of 50$ was paid to all participants.

## 3 Results

### 3.1 Translation and Linguistic Validation

For the translation and linguistic validation panel, four participants were recruited, respectively. Sociodemographic characteristics of the panel members are displayed in Table 1.

**Table 1:** Characteristics of the dual‐panel members.

	N	Age (years) M ± SD (range)	Sex		Background
			Female (N)	Male (N)	
Translation					
	4	55.8 ± 27.22 (27–79)	3	1	2 language teachers (grown up bilingual), 2 Danish native speakers
Linguistic validation					
	4	36.5 ± 19.77 (24–66)	4	—	4 employees

*Note:* M, mean; SD, standard deviation.

When translating the Danish version of the HCUTI into German, the bilingual panel found appropriate wordings for all items, instructions and response options. In the subsequent review of the translated version, the lay panel generally approved the translated version, but discussed the wording of single items and made suggestions for their modification. Overall, consensus could be reached concerning the translated version (Supporting Information S2: Material S1), which was used for the subsequent evaluation of content validity.

### 3.2 Content Validation

Content validity was assessed in three rounds of cognitive interviews including seven women with a history of uUTI and seven experts, respectively. For each interview round, new participants were recruited. In total, 42 women and experts were recruited. The initial version of the HCUTI was examined in the first round (April 29 to May 30, 2023), and modified versions of the questionnaire were evaluated in the second (July 31 to September 15, 2023) and third round (October 14 to 30, 2023) of interviews. Sociodemographic data of the study participants are depicted in Table 2.

**Table 2:** Characteristics of study participants for content validity assessments.

	N	Age (years) M ± SD (range)	Sex		Background
			Female (N)	Male (N)	
**Round 1**					
Women	7	28.9 ± 3.67 (36–34)	7	—	4 employees, 3 students
Experts	7	41.9 ± 15.72 (27–68)	5	2	2 health scientists, 1 general practitioner, 1 urologist, 1 gynaecologist, 1 midwife, 1 statistician
**Round 2**					
Women	7	44.3 ± 21.57 (25–73)	7	—	5 employees, 2 students
Experts	7	56.6 ± 17.74 (25–75)	4	3	3 gynaecologists, 2 urologists, 2 health scientists
**Round 3**					
Women	7	24.7 ± 0.95 (23–26)	7	—	7 students
Experts	7	41.1 ± 11.55 (23–57)	5	2	2 urologists, 1 gynaecologist, 1 nurse, 1 psychologist, 1 statistician, 1 PROM expert

Abbreviations: M, mean; PROM, patient‐reported outcome measure; SD, standard deviation.

#### 3.2.1 First Round of Interviews

##### 3.2.1.1 Relevance

The instructions, response options and recall period were considered appropriate. Participants rated the items generally as relevant, but made important suggestions regarding the content of single items. Arguing that it is difficult to distinguish between certain sensations, some women and experts proposed to merge the following items assessing symptoms:

- Item 1a (“Pain on urination”) and item 2a (“Burning sensation on urination”)
- Item 13a (“Pain around the bladder”) and item 14a (“Uncomfortable pressure around the bladder”)
- Item 15a (“Pain in lower back”) and item 16a (“Uncomfortable pressure in lower back”)

Further, the relevance of item 3a (“Difficulty emptying bladder”), item 10a (“Has to hurry to the toilet”), and item 12a (“Feeling unwell”) was questioned by some women and experts. Acknowledging these concerns, the study team decided to maintain these items, but to explicitly assess their relevance in a second round of interviews. Concerning item 4a (“Smelly urine”) and item 5a (“Urine changed appearance”), participants suggested to add the word “noticeably” to create a stronger contrast to the normal health status. Since the HCUTI is intended to be completed before going to bed, participants preferred to use “this day” instead of “last day” in item 7a assessing daytime frequency of urination. The relevance of item 22 capturing the impact of uUTI on cycling was questioned since cycling is less common in Germany than in Denmark. In agreement with the developer of the HCUTI (AH), this item was discarded.

##### 3.2.1.2 Comprehensiveness

The study participants rated the questionnaire as comprehensive. Single experts made suggestions for extension of the questionnaire, e.g., by assessing the frequency of uUTIs, comorbidities, frequency of medication intake and drinking amount. However, these suggestions were not considered by the study team since such questions would go beyond the scope of the HCUTI.

##### 3.2.1.3 Comprehensibility

Overall, participants considered the wording “has bothered me” instead of “was burdened” for all items assessing bothersomeness of symptoms most appropriate. Some women commented on the wording of the items assessing the bothersomeness of urine odour and appearance (item 4b/5b), and suggested to use the following phrasing: “I was worried about…”. To clarify the appearance of urine, experts proposed to add the following examples to item 5a: dark yellow, cloudy or flaky. With respect to item 17a evaluating fever, some women argued that it would be more appropriate to ask for the presence of higher body temperature or fever rather than for the feeling to have fever. The study team decided in agreement with the developer of the HCUTI (AH) to modify this item accordingly, but to assess its comprehensibility in further interviews. Participants also proposed a minor linguistic modification of item 21 assessing the impact of uUTI on exercise by adding “than usual,” of item 24 assessing the impact of uUTI on sleep by replacing “difficulties to sleep” by “slept badly,” and to replace “desire for sex” by “desire for intimacy” in item 25 assessing the impact of uUTI on sex.

##### 3.2.1.4 Other Comments

Concerning the figure relating to item 13a (“Pain around the bladder”) and 15a (“Pain in lower back”) to display the bladder and lower back area, participants advocated for replacing the image of a male body by an image of a female body.

Based on the results of the first round of content validity assessments, the study team adapted the questionnaire, and the modified version was evaluated in a second round of interviews.

#### 3.2.2 Second Round of Interviews

##### 3.2.2.1 Relevance

Instructions, response options and recall period were considered appropriate. When explicitly addressing the relevance of the items measuring “Pain on urination,” “Burning sensation on urination,” “Pain around the bladder,” “Uncomfortable pressure around the bladder,” “Pain in lower back” and “Uncomfortable pressure in lower back,” study participants preferred to assess these symptoms using single items, respectively. Therefore, the study team decided to retain the initial version of the questionnaire in this regard. The relevance of item 3a (“Difficulty emptying bladder”), 10a (“Has to hurry to the toilet”) and 12a (“Feeling unwell”) was not questioned in the second round of interviews, and these items were also maintained as in the initial version of the questionnaire. However, item 3a (“Difficulty emptying bladder”) was reformulated as follows: “I had the feeling that I couldn't empty my bladder completely.”

##### 3.2.2.2 Comprehensiveness

Study participants considered the questionnaire as comprehensive. Some experts made suggestions for extension of the questionnaire similar to the experts in the first round of interviews, and also their suggestions were not considered by the study team.

##### 3.2.2.3 Comprehensibility

No issues regarding comprehensiblity were noted by the participants. The study team considered the participants’ suggestions for linguistic modification of items 21 and 24 assessing the impact of uUTI on exercise and sex, respectively, of minor relevance, and decided to maintain the item formulation as in the initial version of the questionnaire. All other modifications resulting from the first round of content validity assessments were accepted by the participants.

##### 3.2.2.4 Other Comments

When the study team reviewed the results of the cognitive interviews, concerns regarding the appropriateness of single response options emerged. For the items assessing odour and appearance of urine, blood in urine, daytime and nighttime frequency of urination, increased urge for urination, having to hurry to the toilet, incontinence, fever and shivering, the study team decided to modify the response options in agreement with the developer of the HCUTI (AH). Since it is difficult to rate the severity of these symptoms, a dichotomous scale was introduced to assess whether the respective symptom is present or not. Moreover, for all other items, the response options were linguistically modified as follows: does not apply (0), rather does not apply (1), tends to apply (2) and fully applies (3). Additionally, concerning the items on impact, “no” was replaced by “not relevant”.

Based on the results of the second round of content validity assessments, the questionnaire was modified again, and a third round of cognitive interviews was performed.

#### 3.2.3 Third Round of Interviews

Participants had no comments regarding relevance, comprehensibility and comprehensiveness of the questionnaire, and also approved appropriateness of the instructions, response options and recall period. As a minor modification to enhance comprehensibility, item 17 assessing fever was extended by explicating the measurement value to define an elevated body temperature or fever (> 37.5°C) following the suggestions of the study participants.

The study team reviewed and linguistically standardized the final version of the questionnaire to ensure consistency in wording and sentence structure across all items. The final German version of the HCUTI is displayed in Supporting Information S1: Appendix B.

## 4 Discussion

The Danish version of the HCUTI was successfully translated and adapted into German language using the dual‐panel method. This approach is considered an adequate alternative to the widely used forward‐and‐back‐translation method because it provides a consensus‐based translation and adaptation including lay people, thereby ensuring quality of the translation, acceptability and comprehensibility of the wording and ease of completion during the process [14, 17]. Translation and linguistic validation resulted in a German version of the HCUTI which lay persons considered easy to understand. In content validity assessments, participants rated the questionnaire as generally relevant and comprehensive. One item was removed due to lack of relevance. Further modifications concerned the wording of the response options and single items, and the scaling of ten items on symptoms was revised. After modification, the German version of the HCUTI includes 18 items on symptoms, 18 items on bothersomeness, and six items on impact on daily activities.

A major strength of the present study is the adherence to the recommendations of the COSMIN group [15] in the assessment of content validity. Supporting the development of high‐quality measurement instruments, the COSMIN methodology represents international, research‐based practice in measurement and statistics for instruments in healthcare [18].

The HCUTI was intensively evaluated in three rounds of cognitive interviews including in total 21 women with a history of uUTI and 21 experts from relevant medical specialities, health sciences and research methodology to capture a variety of perspectives. Following modifications across this process, all participants rated the HCUTI as relevant, comprehensible and comprehensive in the final round of interviews, suggesting sufficient content validity of the instrument. As a limitation, decisions regarding potential modifications of the questionnaire were not supported by quantitative analyses of expert ratings, e.g. by calculating the item content validity index (I‐CVI) [19] as indicator of the degree of agreement between the raters. However, numerical ratings informed potential modifications, i.e. if at least three participants stated that an item requires at least minor modifications, this was interpreted as modification issue and subsequently discussed in the research team based on the experts’ concrete statements. Another limitation may arise from the fact that the group of women with a history of uUTI comprised primarily younger women with higher education as reflected by the large proportion of students. Including women with a broader range of age and educational level, but also women with different ethnic background would have been beneficial to investigate the instrument's comprehensibility, but was not feasible despite intensive recruitment efforts.

Given that developing new PROMs is time‐consuming and requires substantial financial resources [20], cross‐cultural adaptation and validation of existing instruments is an appropriate approach to make a specific instrument accessible for use in different culture and language settings [21]. Indicating that the Danish version of the HCUTI is suitable for this purpose, sufficient content validity of this tool has been found in our previous systematic review of the quality of PROMs for women with uUTI [8]. As part of a larger research project aiming to perform translation and cultural adaptation of the HCUTI for use in the German language setting, and to assess content validity and psychometric properties of the German version of the HCUTI [22], our study is the first to use the Danish HCUTI for cross‐cultural adaptation and validation. Reflecting cultural peculiarities, the item assessing the impact of uUTI on cycling was removed in the German version of the HCUTI since this activity was considered of minor relevance in the German culture. To enhance comprehensibility, eight items on symptoms and bothersomeness, and one item on daily activities were slightly reformulated. As a major modification, ten items in the symptoms domain that originally had four response options were dichotomized. Moreover, we revised the response options from the quantification of symptoms, bothersomeness and impact (0 = *none*, 1 = *a little*, 2 = *some*, 3 = *a lot*) to the assessment of agreement (0 = *does not apply, rather does not apply*, 3 = *tends to apply*, 4 = *fully applies*).

With content validity of the German version of the HCUTI being established, analysis of psychometric properties is warranted. In this regard, it is of importance to note that data for comparison is limited. For the Danish version of the HCUTI, structural validity, internal consistency and differential item functioning have been investigated [9]. According to our previous systematic review, the results on structural validity and internal consistency were indeterminate, but we found evidence for no important differential item functioning [8].

Taken together, content validity is considered the most important measurement property of PROMs [23], and our data demonstrate sufficient content validity of the German version of the HCUTI for the assessment of symptoms, bothersomeness and impact of uUTI on daily activities in terms of relevance, comprehensibility and comprehensiveness. Although the overall aim of cross‐cultural validation of PROMs is to maintain content validity [21], our findings emphasize the necessity to check whether all items are relevant for the measurement of the intended construct in the target culture. To provide comprehensive evidence about the measurement validity of the German version of the HCUTI, data on the psychometric properties of the instrument will be collected in a subsequent survey among women with uUTI. In this regard, testing for unidimensionality of the three proposed domains and analyses on differential item functioning are of particular importance. In view of potential application of the HCUTI in future studies, also aspects of acceptability and feasibility will be investigated.

## Ethics Statement

The study was approved by the Ethics Committee of the Otto‐von‐Guericke University, Medical Faculty and University Hospital Magdeburg (No. 19/23).

## Consent

All study participants gave written informed consent.

## Conflicts of Interest

Christian Apfelbacher received institutional funding from Bionorica SE and Dr Wolf Group and consultancy fees from Bionorica SE, Dr Wolff Group, Rheacell, Sanofi, Pfizer, LEOPharma and Incyte for services related to patient‐reported outcome measures. Anne Holm received funding for research from Allergica A/S, a company producing homeopathic medicine. The other authors declare no conflicts of interest.

## Supporting information

HCUTI Content validation appendices revised.

HCUTI Content validation supplement revised.

## Data Availability

The data that support the findings of this study are available on request from the corresponding author. The data are not publicly available due to privacy or ethical restrictions.
