# Supplementary material for: The Holm and Cordoba Urinary Tract Infection Score: Translation, Linguistic and Content Validation of the German Version of a Patient‐Reported Outcome Measure to Assess Symptoms, Bothersomeness and Impact of Uncomplicated Urinary Tract Infections in Women
Source: Neurourol Urodyn. 2025 May 8;44(5):1064–70. doi: 10.1002/nau.70066 (PMC12164243; doi:10.1002/nau.70066)
Supplement: Supplementary file 1 — HCUTI Content validation appendices revised. [file NAU-44-1064-s001.docx]

**Appendix A: Cognitive interview guide**

**Welcoming**

Thank you for taking the time for the interview. With the interview, you are supporting a research project of the Institute for Social Medicine and Health Systems Research. In this project, we aim to evaluate a questionnaire assessing symptoms and impact of acute uncomplicated urinary tract infections in women. For this purpose, we would like to discuss with you whether the items are relevant and comprehensive. We have invited you for this interview since you have experienced an uncomplicated urinary tract infection in the past, and your experiences are a valuable contribution to the evaluation and potential modification of the questionnaire. The interview will probably last for about 60 minutes.

**Instruction**

Before we start, it is important for me to mention that there are no right or wrong answers. For this interview, your opinion and your experiences are crucial. I will lead the conversation. We will evaluate the questionnaire item-by-item using the criteria I have sent to you. Please open the file to you have it in front of you all the time. Alternatively, you can write the criteria down or print the file out.

| **Comprehensibility** | |  | **Relevance** | |
| --- | --- | --- | --- | --- |
| **Code** | **Meaning** |  | **Code** | **Meaning** |
| 1 | Wording is clear |  | 1 | Activity is relevant |
| 2 | Must be presented slightly different |  | 2 | Must be presented slightly different |
| 3 | Must be presented clearly different |  | 3 | Must be presented clearly different |
| 4 | Wording is not clear |  | 4 | Activity is not relevant |

For each item, we will assess the comprehensibility of the wording and the relevance of its content. Please indicate whether the item is clearly understandable for you, or whether you consider minor or major changes necessary to understand the item better. Likewise, we will evaluate the relevance of each item.

We would like to record the interview for later analysis. Your data will be stored and analyzed anonymously with no linkage to your person. If you need a break during the interview, just let me know anytime. If you agree, I would start the record now.

**Assessment**

Note: In addition to the evaluation of the questionnaire according to the predefined criteria, participants are welcome to make suggestions for alternative wording or content regarding the instructions, items, response options and recall period.

*General instruction:*

At the beginning, I would like to ask you to read the instruction for the questionnaire aloud.

- How clear is the instruction for you?
- Are there any sentences that you consider not relevant?

*Comprehensibility (item-by-item):*

- Please read the sentence aloud. With regard to the response notions noted for comprehensibility and clarity, how would you rate this item?

*Relevance (item-by-item):*

- How relevant is this item for you? Here, too, you may use the defined response options.

*Recall period und response options (assessed after the evaluation of all items):*

- In retrospect, all questions refer to the last 24 hours. Do you consider this recall period appropriate?
- Let’s take a closer look at the response options. Do you consider the response options appropriate and understandable?

*Overall impression:*

- What is your overall impression of the questionnaire?
- Are there important aspects you think that are missing in the questionnaire?
- Do you have any suggestions for improving the questionnaire?

*Summary:*

- Is there anything else you would like to add to our conversation? Perhaps an aspect that has not been mentioned so far, but you consider important?
- The interview is now finished. Thank you very much for taking the time to meet with me and I wish you all the best.

Appendix B: Final German version of the Holm and Cordoba Urinary Tract Infection Score (HCUTI)


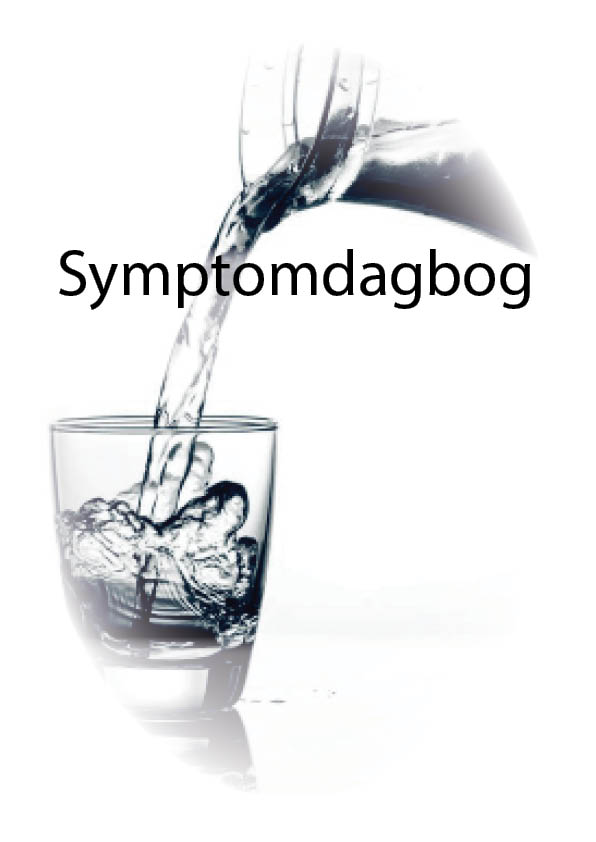


Symptomtagebuch

**Fragen zu Symptomen und Beeinträchtigungen**

Bitte geben Sie an, in welchem Ausmaß Sie in den letzten 24 Stunden folgende Symptome hatten und wie sehr diese Sie beeinträchtigt haben.

|  |  | **Trifft nicht zu** | **Trifft eher nicht zu** | **Trifft**  **eher zu** | **Trifft voll zu** | **Weiß nicht** |
| --- | --- | --- | --- | --- | --- | --- |
| 1a | Ich hatte in den letzten 24 Stunden Schmerzen beim Wasserlassen. | 🞎 | 🞎 | 🞎 | 🞎 | 🞎 |
| 1b | *Die Schmerzen beim Wasserlassen haben mich beeinträchtigt.* | 🞎 | 🞎 | 🞎 | 🞎 | 🞎 |
| 2a | Ich hatte in den letzten 24 Stunden ein Brennen beim Wasserlassen. | 🞎 | 🞎 | 🞎 | 🞎 | 🞎 |
| 2b | *Das Brennen beim Wasserlassen hat mich beeinträchtigt.* | 🞎 | 🞎 | 🞎 | 🞎 | 🞎 |
| 3a | Ich hatte in den letzten 24 Stunden das Gefühl, die Blase nicht vollständig entleeren zu können. | 🞎 | 🞎 | 🞎 | 🞎 | 🞎 |
| 3b | *Das Gefühl, die Blase nicht vollständig entleeren zu können, hat mich beeinträchtigt.* | 🞎 | 🞎 | 🞎 | 🞎 | 🞎 |
| 4a | Mein Urin hat in den letzten 24 Stunden auffällig anders gerochen als sonst. | **Nein** | | **Ja** | | **Weiß nicht** |
|  |  | 🞎 | | 🞎 | | 🞎 |
| 4b | *Es hat mir Sorgen bereitet, dass mein Urin auffällig anders gerochen hat als sonst.* | **Trifft nicht zu** | **Trifft eher nicht zu** | **Trifft**  **eher zu** | **Trifft voll zu** | **Weiß nicht** |
|  |  | 🞎 | 🞎 | 🞎 | 🞎 | 🞎 |
| 5a | Mein Urin hat in den letzten 24 Stunden auffällig anders ausgesehen (z.B. dunkelgelb, trüb, oder flockig) als sonst. | **Nein** | | **Ja** | | **Weiß nicht** |
|  |  | 🞎 | 🞎 | 🞎 | 🞎 | 🞎 |
| 5b | *Es hat mir Sorgen bereitet, dass mein Urin auffällig anders ausgesehen hat als sonst.* | **Trifft nicht zu** | **Trifft eher nicht zu** | **Trifft**  **eher zu** | **Trifft voll zu** | **Weiß nicht** |
|  |  | 🞎 | 🞎 | 🞎 | 🞎 | 🞎 |
| 6a | Ich habe in den letzten 24 Stunden Blut im Urin gesehen. | **Nein** | | **Ja** | | **Weiß nicht** |
|  |  | 🞎 | 🞎 | 🞎 | 🞎 | 🞎 |
| 6b | *Es hat mir Sorgen bereitet, Blut im Urin zu sehen.* | **Trifft nicht zu** | **Trifft eher nicht zu** | **Trifft**  **eher zu** | **Trifft voll zu** | **Weiß nicht** |
|  |  | 🞎 | 🞎 | 🞎 | 🞎 | 🞎 |

| 7a | Ich musste am heutigen Tag häufiger Wasserlassen als sonst. | **Nein** | | **Ja** | | **Weiß nicht** |
| --- | --- | --- | --- | --- | --- | --- |
|  |  | 🞎 | 🞎 | 🞎 | 🞎 | 🞎 |
| 7b | *Es hat mich beeinträchtigt, am Tag häufiger Wasserlassen zu müssen als sonst.* | **Trifft nicht zu** | **Trifft eher nicht zu** | **Trifft**  **eher zu** | **Trifft voll zu** | **Weiß nicht** |
|  |  | 🞎 | 🞎 | 🞎 | 🞎 | 🞎 |
| 8a | Ich musste in der vergangenen Nacht häufiger Wasserlassen als sonst. | **Nein** | | **Ja** | | **Weiß nicht** |
|  |  | 🞎 | | 🞎 | | 🞎 |
| 8b | *Es hat mich beeinträchtigt, in der Nacht häufiger Wasserlassen zu müssen als sonst.* | 🞎 | 🞎 | 🞎 | 🞎 | 🞎 |
| 9a | Ich habe in den letzten 24 Stunden mehr Harndrang empfunden als sonst. | **Nein** | | **Ja** | | **Weiß nicht** |
|  |  | 🞎 | | 🞎 | | 🞎 |
| 9b | *Es hat mich beeinträchtigt, mehr Harndrang zu empfinden.* | **Trifft nicht zu** | **Trifft eher nicht zu** | **Trifft**  **eher zu** | **Trifft voll zu** | **Weiß nicht** |
|  |  | 🞎 | 🞎 | 🞎 | 🞎 | 🞎 |
| 10a | Ich habe mich in den letzten 24 Stunden zum Wasserlassen zur Toilette beeilen müssen. | **Nein** | | **Ja** | | **Weiß nicht** |
|  |  | 🞎 | | 🞎 | | 🞎 |
| 10b | *Es hat mich beeinträchtigt, mich zur Toilette beeilen zu müssen.* | **Trifft nicht zu** | **Trifft eher nicht zu** | **Trifft**  **eher zu** | **Trifft voll zu** | **Weiß nicht** |
|  |  | 🞎 | 🞎 | 🞎 | 🞎 | 🞎 |
| 11a | Ich hatte in den letzten 24 Stunden Schwierigkeiten das Wasser zu halten. | **Nein** | | **Ja** | | **Weiß nicht** |
|  |  | 🞎 | | 🞎 | | 🞎 |
| 11b | *Die Schwierigkeiten, das Wasser zu halten, haben mich beeinträchtigt.* | **Trifft nicht zu** | **Trifft eher nicht zu** | **Trifft**  **eher zu** | **Trifft voll zu** | **Weiß nicht** |
|  |  | 🞎 | 🞎 | 🞎 | 🞎 | 🞎 |
| 12a | Ich habe mich in den letzten 24 Stunden unwohl gefühlt. | 🞎 | 🞎 | 🞎 | 🞎 | 🞎 |
| 12b | *Mich unwohl zu fühlen, hat mich beeinträchtigt.* | 🞎 | 🞎 | 🞎 | 🞎 | 🞎 |
| 13a | Ich hatte in den letzten 24 Stunden Schmerzen im Blasenbereich (siehe Abbildung). | 🞎 | 🞎 | 🞎 | 🞎 | 🞎 |
| 13b | *Es hat mich beeinträchtigt, im Blasenbereich Schmerzen zu haben.* | 🞎 | 🞎 | 🞎 | 🞎 | 🞎 |

|  |  | **Trifft nicht zu** | **Trifft eher nicht zu** | **Trifft**  **eher zu** | **Trifft voll zu** | **Weiß nicht** |
| --- | --- | --- | --- | --- | --- | --- |
| 14a | Ich hatte in den letzten 24 Stunden ein unangenehmes inneres Druckgefühl im Blasenbereich. | 🞎 | 🞎 | 🞎 | 🞎 | 🞎 |
| 14b | *Es hat mich beeinträchtigt, ein unangenehmes inneres Druckgefühl im Blasenbereich zu haben.* | 🞎 | 🞎 | 🞎 | 🞎 | 🞎 |
| 15a | Ich hatte in den letzten 24 Stunden Schmerzen im Nierenbereich (siehe Abbildung). | 🞎 | 🞎 | 🞎 | 🞎 | 🞎 |
| 15b | *Es hat mich beeinträchtigt, Schmerzen im Nierenbereich zu haben.* | 🞎 | 🞎 | 🞎 | 🞎 | 🞎 |
| 16a | Ich hatte in den letzten 24 Stunden ein unangenehmes inneres Druckgefühl im Nierenbereich (siehe Abbildung). | 🞎 | 🞎 | 🞎 | 🞎 | 🞎 |
| 16b | *Es hat mich beeinträchtigt, ein unangenehmes inneres Druckgefühl im Nierenbereich zu haben.* | 🞎 | 🞎 | 🞎 | 🞎 | 🞎 |
| 17a | Ich hatte in den letzten 24 Stunden erhöhte Temperatur oder Fieber  (>37,5°C). | **Nein** | | **Ja** | | **Weiß nicht** |
|  |  | 🞎 | | 🞎 | | 🞎 |
| 17b | *Es hat mich beeinträchtigt, erhöhte Temperatur oder Fieber zu haben.* | **Trifft nicht zu** | **Trifft eher nicht zu** | **Trifft**  **eher zu** | **Trifft voll zu** | **Weiß nicht** |
|  |  | 🞎 | 🞎 | 🞎 | 🞎 | 🞎 |
| 18a | Ich hatte in den letzten 24 Stunden Schüttelfrost. | **Nein** | | **Ja** | | **Weiß nicht** |
|  |  | 🞎 | | 🞎 | | 🞎 |
| 18b | *Es hat mich beeinträchtigt, Schüttelfrost zu haben.* | **Trifft nicht zu** | **Trifft eher nicht zu** | **Trifft**  **eher zu** | **Trifft voll zu** | **Weiß nicht** |
|  |  | 🞎 | 🞎 | 🞎 | 🞎 | 🞎 |


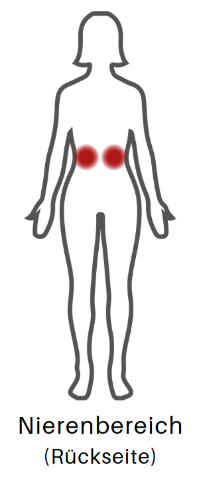

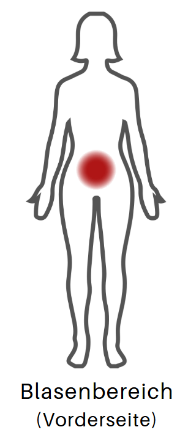


**Fragen zu Auswirkungen der Erkrankung**

Bitte geben Sie an, welche Tätigkeiten Ihnen in den letzten 24 Stunden aufgrund Ihrer Blasenentzündung Schwierigkeiten bereitet haben.

|  |  | **Nicht relevant** | **Trifft nicht zu** | **Trifft eher nicht zu** | **Trifft eher zu** | **Trifft voll zu** |
| --- | --- | --- | --- | --- | --- | --- |
| 19 | Es fiel mir schwer, in den letzten 24 Stunden meine Arbeit oder vergleichbare Tätigkeiten zu erledigen. |  | 🞎 | 🞎 | 🞎 | 🞎 |
| 20 | Es fiel mir schwer, in den letzten 24 Stunden an sozialen Aktivitäten teilzunehmen. |  | 🞎 | 🞎 | 🞎 | 🞎 |
| 21 | Es fiel mir schwer, in den letzten 24 Stunden Sport zu treiben. | 🞎 | 🞎 | 🞎 | 🞎 | 🞎 |
| 22 | Ich hatte in den letzten 24 Stunden Schwierigkeiten, Arbeiten im Haushalt zu erledigen. |  | 🞎 | 🞎 | 🞎 | 🞎 |
| 23 | Ich habe in den letzten 24 Stunden nicht gut geschlafen. |  | 🞎 | 🞎 | 🞎 | 🞎 |
| 24 | Ich hatte in den letzten 24 Stunden weniger Lust auf Sex. | 🞎 | 🞎 | 🞎 | 🞎 | 🞎 |
