# Supplementary material for: The Holm and Cordoba Urinary Tract Infection Score: Translation, Linguistic and Content Validation of the German Version of a Patient‐Reported Outcome Measure to Assess Symptoms, Bothersomeness and Impact of Uncomplicated Urinary Tract Infections in Women
Source: Neurourol Urodyn. 2025 May 8;44(5):1064–70. doi: 10.1002/nau.70066 (PMC12164243; doi:10.1002/nau.70066)
Supplement: Supplementary file 2 — HCUTI Content validation supplement revised. [file NAU-44-1064-s002.docx]

Supplementary material: Translated German version of the Holm and Cordoba Urinary Tract Infection Score (HCUTI)


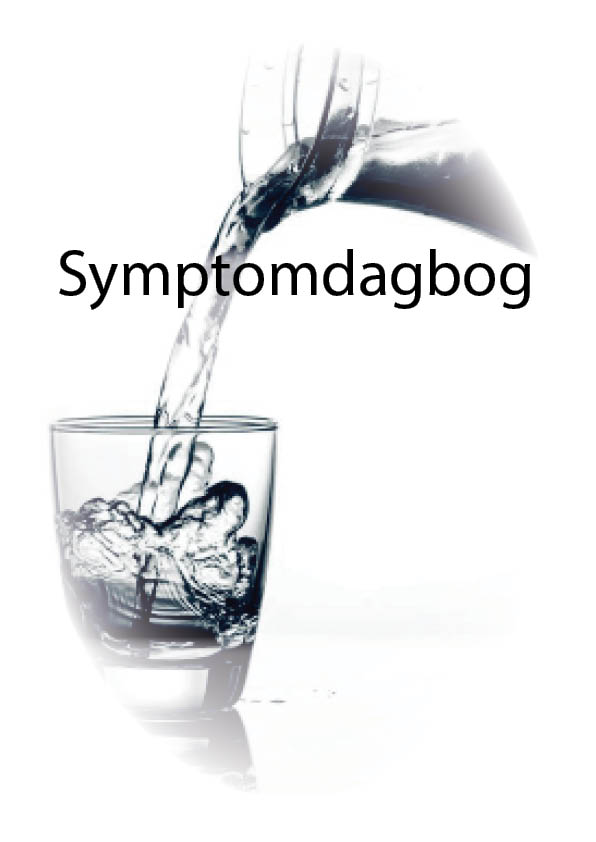


Symptomtagebuch

**Fragen zu Symptomen und Beeinträchtigungen**

Bitte geben Sie an, in welchem Ausmaß Sie in den letzten 24 Stunden folgende Symptome hatten und wie sehr diese Sie beeinträchtigt haben.

|  |  | **Nein** | **Ja,**  **wenig** | **Ja,**  **etwas** | **Ja,**  **sehr** | **Weiß**  **nicht** |
| --- | --- | --- | --- | --- | --- | --- |
| 1a | Ich habe in den letzten 24 Stunden Schmerzen beim Wasserlassen gehabt. | 🞎 | 🞎 | 🞎 | 🞎 | 🞎 |
| 1b | *Die Schmerzen beim Wasserlassen haben mich belastet.* | 🞎 | 🞎 | 🞎 | 🞎 | 🞎 |
| 2a | Ich habe in den letzten 24 Stunden ein Brennen beim Wasserlassen gehabt. | 🞎 | 🞎 | 🞎 | 🞎 | 🞎 |
| 2b | *Das Brennen beim Wasserlassen hat mich belastet.* | 🞎 | 🞎 | 🞎 | 🞎 | 🞎 |
| 3a | Ich habe in den letzten 24 Stunden Schwierigkeiten gehabt, die Blase zu entleeren. | 🞎 | 🞎 | 🞎 | 🞎 | 🞎 |
| 3b | *Die Schwierigkeiten, die Blase zu entleeren, haben mich belastet.* | 🞎 | 🞎 | 🞎 | 🞎 | 🞎 |
| 4a | In den letzten 24 Stunden hat mein Urin anders gerochen als sonst. | 🞎 | 🞎 | 🞎 | 🞎 | 🞎 |
| 4b | *Es hat mich beeinträchtigt, dass mein Urin anders gerochen hat als sonst.* | 🞎 | 🞎 | 🞎 | 🞎 | 🞎 |
| 5a | Mein Urin hat in den letzten 24 Stunden anders ausgesehen als sonst. | 🞎 | 🞎 | 🞎 | 🞎 | 🞎 |
| 5b | *Es hat mich beeinträchtigt, dass mein Urin anders ausgesehen hat als sonst.* | 🞎 | 🞎 | 🞎 | 🞎 | 🞎 |
| 6a | Ich habe in den letzten 24 Stunden Blut im Urin gesehen. | 🞎 | 🞎 | 🞎 | 🞎 | 🞎 |
| 6b | *Es hat mir Sorgen bereitet, Blut im Urin zu sehen.* | 🞎 | 🞎 | 🞎 | 🞎 | 🞎 |
| 7a | Ich musste am vergangenen Tag häufiger Wasserlassen als sonst. | 🞎 | 🞎 | 🞎 | 🞎 | 🞎 |
| 7b | *Es hat mich beeinträchtigt, häufiger am Tag Wasserlassen zu müssen.* | 🞎 | 🞎 | 🞎 | 🞎 | 🞎 |
| 8a | Ich musste in der vergangenen Nacht häufiger Wasserlassen als sonst. | 🞎 | 🞎 | 🞎 | 🞎 | 🞎 |
| 8b | *Es hat mich beeinträchtigt, in der Nacht häufiger Wasserlassen zu müssen.* | 🞎 | 🞎 | 🞎 | 🞎 | 🞎 |
| 9a | Ich habe in den letzten 24 Stunden mehr Harndrang empfunden als sonst. | 🞎 | 🞎 | 🞎 | 🞎 | 🞎 |
| 9b | *Mehr Harndrang zu empfinden, hat mich belastet.* | 🞎 | 🞎 | 🞎 | 🞎 | 🞎 |
| 10a | Ich habe mich in den letzten 24 Stunden zum Wasserlassen zur Toilette beeilen müssen. | 🞎 | 🞎 | 🞎 | 🞎 | 🞎 |
| 10b | *Mich zur Toilette beeilen zu müssen, hat mich belastet.* | 🞎 | 🞎 | 🞎 | 🞎 | 🞎 |

|  |  | **Nein** | **Ja,**  **wenig** | **Ja,**  **etwas** | **Ja,**  **sehr** | **Weiß**  **nicht** |
| --- | --- | --- | --- | --- | --- | --- |
| 11a | Ich habe in den letzten 24 Stunden Schwierigkeiten gehabt das Wasser zu halten. | 🞎 | 🞎 | 🞎 | 🞎 | 🞎 |
| 11b | *Die Schwierigkeiten, zu haben das Wasser zu halten, haben mich belastet.* | 🞎 | 🞎 | 🞎 | 🞎 | 🞎 |
| 12a | Ich habe mich in den letzten 24 Stunden unwohl gefühlt. | 🞎 | 🞎 | 🞎 | 🞎 | 🞎 |
| 12b | *Mich unwohl zu fühlen hat mich belastet.* | 🞎 | 🞎 | 🞎 | 🞎 | 🞎 |
| 13a | Ich hatte in den letzten 24 Stunden Schmerzen im Blasenbereich (siehe Abbildung). | 🞎 | 🞎 | 🞎 | 🞎 | 🞎 |
| 13b | *Es hat mich beeinträchtigt, im Blasenbereich Schmerzen zu haben.* | 🞎 | 🞎 | 🞎 | 🞎 | 🞎 |
| 14a | Ich habe in den letzten 24 Stunden ein unangenehmes inneres Druckgefühl im Blasenbereich gehabt. | 🞎 | 🞎 | 🞎 | 🞎 | 🞎 |
| 14b | *Es hat mich beeinträchtigt, ein unangenehmes inneres Druckgefühl im Blasenbereich zu haben.* | 🞎 | 🞎 | 🞎 | 🞎 | 🞎 |
| 15a | Ich habe in den letzten 24 Stunden Schmerzen im Nierenbereich gehabt (siehe Abbildung). | 🞎 | 🞎 | 🞎 | 🞎 | 🞎 |
| 15b | *Es hat mich beeinträchtigt, Schmerzen im Nierenbereich zu haben.* | 🞎 | 🞎 | 🞎 | 🞎 | 🞎 |
| 16a | Ich habe in den letzten 24 Stunden ein unangenehmes inneres Druckgefühl im Nierenbereich gehabt (siehe Abbildung). | 🞎 | 🞎 | 🞎 | 🞎 | 🞎 |
| 16b | *Es hat mich beeinträchtigt, ein unangenehmes inneres Druckgefühl im Nierenbereich zu haben.* | 🞎 | 🞎 | 🞎 | 🞎 | 🞎 |
| 17a | Ich hatte in den letzten 24 Stunden das Gefühl Fieber zu haben. | 🞎 | 🞎 | 🞎 | 🞎 | 🞎 |
| 17b | *Es hat mich beeinträchtigt, das Gefühl von Fieber zu haben.* | 🞎 | 🞎 | 🞎 | 🞎 | 🞎 |
| 18a | Ich hatte in den letzten 24 Stunden Schüttelfrost. | 🞎 | 🞎 | 🞎 | 🞎 | 🞎 |
| 18b | *Schüttelfrost zu haben, hat mich beeinträchtigt.* | 🞎 | 🞎 | 🞎 | 🞎 | 🞎 |

**
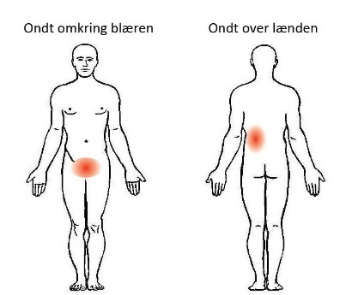
**

Nierenbereich

Blasenbereich

**Fragen zu Auswirkungen der Erkrankung**

Bitte geben Sie an, welche Tätigkeiten Ihnen in den letzten 24 Stunden aufgrund Ihrer Blasenentzündung Schwierigkeiten bereitet haben.

|  |  | **Nicht relevant** | **Ja,**  **wenig** | **Ja,**  **etwas** | **Ja,**  **sehr** | **Weiß**  **nicht** |
| --- | --- | --- | --- | --- | --- | --- |
| 19 | Es fiel mir schwer, in den letzten 24 Stunden meine Arbeit oder vergleichbare Tätigkeiten zu erledigen. | 🞎 | 🞎 | 🞎 | 🞎 | 🞎 |
| 20 | Es fiel mir schwer, in den letzten 24 Stunden an sozialen Aktivitäten teilzunehmen. | 🞎 | 🞎 | 🞎 | 🞎 | 🞎 |
| 21 | Es fiel mir schwer, in den letzten 24 Stunden Sport zu treiben. | 🞎 | 🞎 | 🞎 | 🞎 | 🞎 |
| 22 | Es fiel mir schwer, in den letzten 24 Stunden Fahrrad zu fahren. | 🞎 | 🞎 | 🞎 | 🞎 | 🞎 |
| 23 | Ich hatte in den letzten 24 Stunden Schwierigkeiten, Arbeiten im Haushalt zu erledigen. | 🞎 | 🞎 | 🞎 | 🞎 | 🞎 |
| 24 | Ich hatte in den letzten 24 Stunden Schwierigkeiten gut zu schlafen. | 🞎 | 🞎 | 🞎 | 🞎 | 🞎 |
| 25 | Ich hatte in den letzten 24 Stunden weniger Lust auf Sex. | 🞎 | 🞎 | 🞎 | 🞎 | 🞎 |
